# The effect of phenotyping, adult selection, and mating strategies on genetic gain and rate of inbreeding in black soldier fly breeding programs

**DOI:** 10.1186/s12711-024-00938-y

**Published:** 2024-11-04

**Authors:** Margot Slagboom, Hanne Marie Nielsen, Morten Kargo, Mark Henryon, Laura Skrubbeltrang Hansen

**Affiliations:** 1 Center for Quantitative Genetics and Genomics, Aarhus University, C. F. Møllers Allé 3, 8000 Aarhus, Denmark; 2 VikingGenetics, Ebeltoftvej 16, 8960 Randers SØ, Denmark; 3 Danish Agriculture and Food Council, Axeltorv 3, 1609 Copenhagen, Denmark; 4 Department of Biology, Aarhus University, Ny Munkegade 114-116, 8000 Aarhus, Denmark

## Abstract

**Background:**

The aim of this study was to compare genetic gain and rate of inbreeding for different mass selection breeding programs with the aim of increasing larval body weight (LBW) in black soldier flies. The breeding programs differed in: (1) sampling of individuals for phenotyping (either random over the whole population or a fixed number per full sib family), (2) selection of adult flies for breeding (based on an adult individual’s phenotype for LBW or random from larvae preselected based on LBW), and (3) mating strategy (mating in a group with unequal male contributions or controlled between two females and one male). In addition, the numbers of phenotyped and preselected larvae were varied. The sex of an individual was unknown during preselection and females had higher LBW, resulting in more females being preselected.

**Results:**

Selecting adult flies based on their phenotype for LBW increased genetic gain by 0.06 genetic standard deviation units compared to randomly selecting from the preselected larvae. Fixing the number of phenotyped larvae per family increased the rate of inbreeding by 0.15 to 0.20% per generation. Controlled mating compared to group mating decreased the rate of inbreeding by 0.02 to 0.03% per generation. Phenotyping more than 4000 larvae resulted in a lack of preselected males due to the sexual dimorphism. Preselecting both too few and too many larvae could negatively impact genetic gain, depending on the breeding program.

**Conclusions:**

A mass selection breeding programs in which the adult fly is selected based on their larval phenotype, breeding animals mate in a group and sampling larvae for phenotyping at random over the whole population is recommended for black soldier flies, considering the positive effect on rates of genetic gain and inbreeding. The number of phenotyped and preselected larvae should be calculated based on the expected female weight deviation to ensure sufficient male and female candidates are selected.

**Supplementary Information:**

The online version contains supplementary material available at 10.1186/s12711-024-00938-y.

## Background

Black soldier fly (BSF) or Hermetia illucens is a wasp-like fly belonging to the Stratiomyidae family. Interest in BSF production for feed has increased greatly in recent years [1]. Many insect species have similar protein and essential amino acid contents as soy or fish meal but their production has a smaller environmental impact [2] and they have excellent feed conversion ratios [3]. In addition, BSF as feed reduces competition with human food and can be produced on waste streams of other food production systems [4]. Together, these aspects make BSF production a competitive and more sustainable form of feed production than the use of traditional feed sources.

**Fig. 1:**
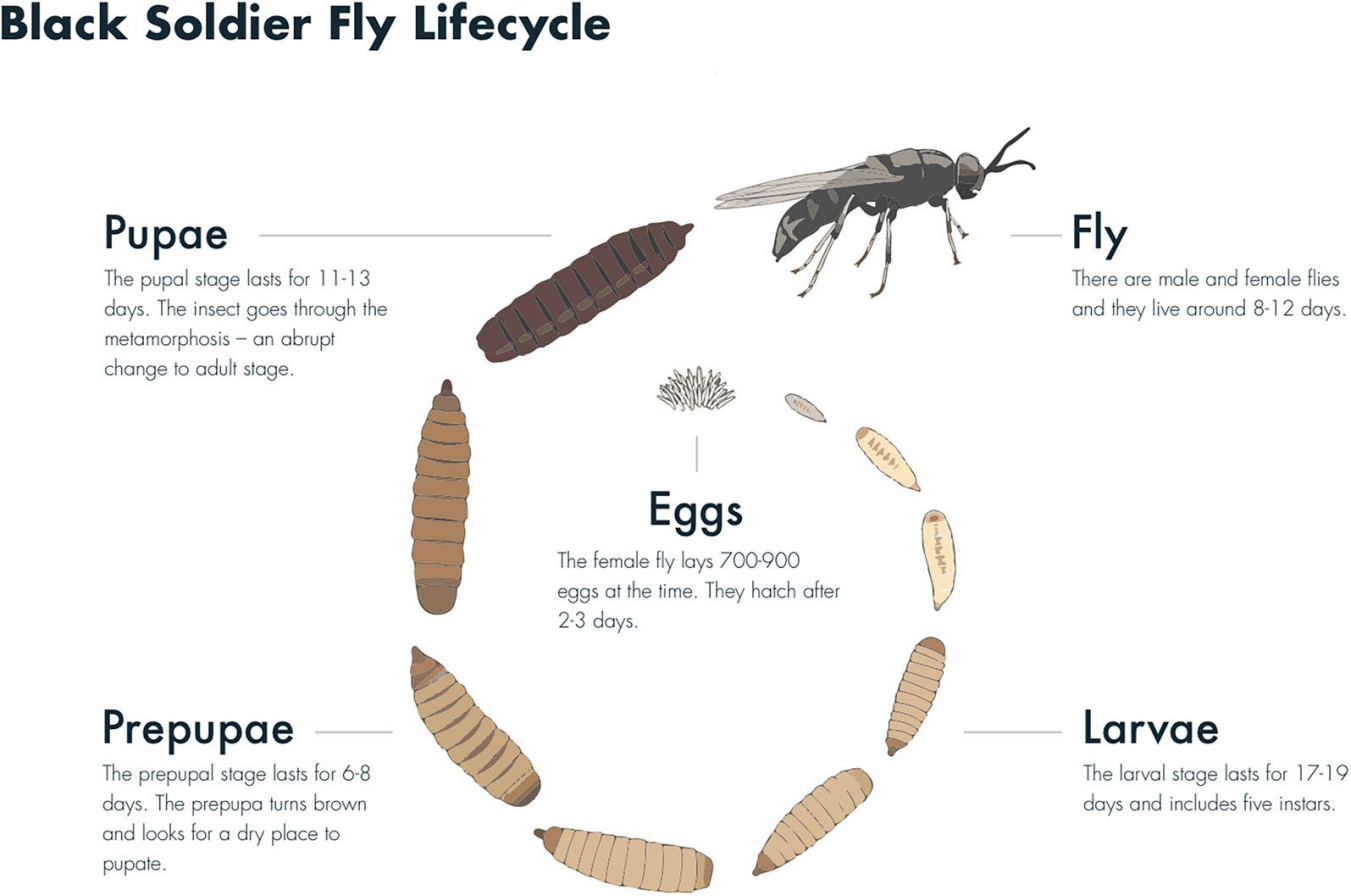
Life cycle of the black soldier fly in industrial production settings [5]

The life cycle of BSF consists of different stages (Fig. 1 [5]). Females lay many eggs that hatch into larvae after 2 to 3 days. The number of eggs laid per female is reported to vary between 205 and 1505 [1]. Larvae feed on an organic substrate until they pupate. Around the prepupal life stage, BSF are the heaviest and contain about 40% protein and 24 to 28% fat [6]. Development time from egg to prepupa can vary between 17 and 19 days in industrial settings but are more extreme in other conditions (17.7 to 32.8 days [1]), depending on, e.g., temperature [7], diet [8], or strain [9]. When flies eclose from the pupae (emerge as adults), sex identification is enabled by visual observation. Reproduction happens at the adult stage and males mate with one or more females (Personal communication, K. Jensen, Aarhus University). In commercial BSF production, most individuals are harvested at the larval or prepupal stage, and only a part of the population is allowed to reproduce. The harvested larvae are ground to a meal, which is the main product of BSF for feed.

Selective breeding can improve chosen traits in the desired direction, as for example shown by [10] for improving body mass in mealworms and by [11] for improving morphometric shape in houseflies. Mass selection is selection on own phenotypic performance, i.e. something that is measurable on the individual itself [12]. It is the simplest way of selective breeding, as it does not require tracking of the pedigree of an individual. This allows for group housing of individuals, which is an advantage in many production systems. Mass selection breeding programs are applied in some aquaculture species for this reason [13]. The short generation interval of BSF enables genetic gain to be accumulated very quickly. Results of the commercial breeding program described in [14] show an impressive improvement in larval body weight by 39% over 10 generations, but details of the breeding program and the applied selection strategies were not presented. Breeding programs consist of various elements, which each can affect rates of genetic gain and inbreeding. These elements can also increase the complexity of the breeding program and, consequently, the amount of labor and level of financial investment required. An example of this is the number of individuals that is phenotyped. Phenotyping more individuals increases the number of selection candidates in a mass selection scheme and thus the ability to select high-performing animals. However, obtaining phenotypic records on many individuals requires intensive manual labor within a limited time period. For the trait larval body weight (LBW), all individuals must be weighted at the same age since hatching, thus at the same day for a synchronized population. Automated phenotyping techniques such as the method described by [15] can greatly decrease labor required but can entail significant expenses. Another example of disparities in complexity is the selection of adult BSF for breeding, which is ideally based on phenotypic information of the trait under selection in a mass selection breeding program. This means selecting adult flies on their larval phenotype when the breeding goal trait is LBW, which is only possible when flies are housed individually after weighing, instead of housing them in a group. When housed in a group, it is impossible to mark and track individuals due to the metamorphic life cycle, in which larvae shed their exoskeleton and eventually pupate. Although it is possible to select BSF already at the larval stages for LBW, due to the sexual dimorphism of body weight in this life stage, more females will be selected, resulting in a lack of male selection candidates. Controlled mating is more complex than mating all animals in a group together but enables control of inbreeding by limiting the contribution from each animal to the next generation. Before implementing them in a BSF breeding program, it is important to quantify the effect of strategies related to, e.g., phenotyping, selection of adult flies for breeding, and mating on rates of genetic gain and inbreeding. Stochastic simulation can be used to predict rates of genetic gain and inbreeding realized by different selection strategies and provide guidelines when establishing a selective breeding program in a large-scale commercial BSF population. In this paper, we hypothesized that breeding programs in which adult flies are selected based on their phenotype for LBW result in greater genetic gain than breeding programs in which flies are randomly selected from a group of larvae preselected for LBW. We also hypothesized that breeding programs in which a fixed number of larvae are phenotyped per family and matings are controlled have lower rates of inbreeding. We tested these hypotheses by comparing genetic gain for LBW and rate of inbreeding for mass selection breeding programs that differ in phenotyping, selection of adult flies for breeding, and mating strategies.

## Methods

**Fig. 2:**
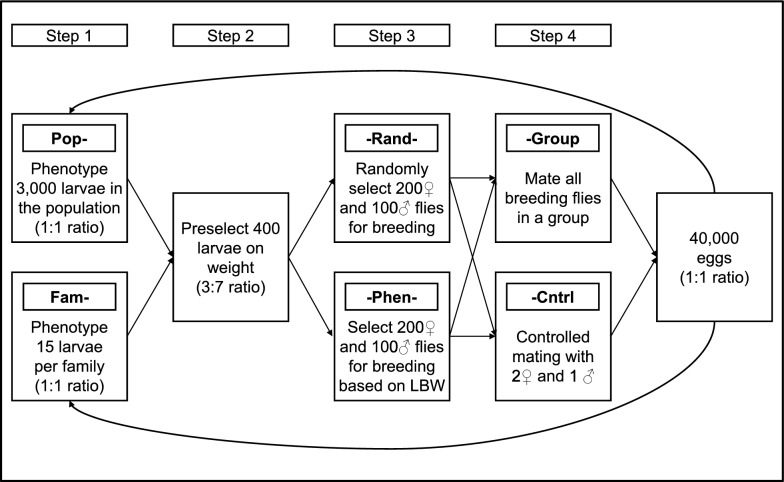
Schematic representation of the breeding schemes with the different selection steps and 3000 phenotyped and 400 preselected larvae. *LBW* Larval body weight. *Ratio* sex ratio

We simulated several breeding programs that differed in three key elements within the breeding scheme: (1) phenotyping either a random sample of larvae across the entire population (Pop- scenarios) or a fixed number of individuals per family (Fam- scenarios); (2) being able to select adult flies for breeding based on their phenotypic record for LBW (-Phen- scenarios) or select them at random (-Rand- scenarios); and (3) mating all breeding animals in a group (-Group scenarios) or controlled mating with a 1:2 male to female ratio (-Cntrl scenarios; Table 1 and Fig. 2). These three key elements together formed the breeding program design. In addition to these elements, the numbers of phenotyped and preselected larvae were varied. All breeding programs were based on mass selection, i.e. own phenotypic performance, for LBW. LBW was assumed to have additive genetic variance 1.0, heritability 0.32, and common environmental variance 0.43 [16]. The common environmental effect was only present in the Fam- scenarios due to the housing of eggs and larvae in full sib groups in these scenarios. Genetic gain was measured in genetic standard deviation units. Each scenario was simulated for 10 time steps which equaled 10 generations. The stochastic simulation program ADAM [17] was used to simulate 100 replicates per scenario.

**Table 1:** The simulated breeding schemes

Breeding scheme	Phenotyping strategy (Step 1^a^)	Selection of flies (Step 3^b^)	Mating strategy (Step 4^c^)
Pop-Rand-Group	Population	Random	Group
Pop-Rand-Cntrl	Population	Random	Controlled
Pop-Phen-Group	Population	Phenotype for LBW^d^	Group
Pop-Phen-Cntrl	Population	Phenotype for LBW	Controlled
Fam-Rand-Group	Fixed number per family	Random	Group
Fam-Rand-Cntrl	Fixed number per family	Random	Controlled
Fam-Phen-Group	Fixed number per family	Phenotype for LBW	Group
Fam-Phen-Cntrl	Fixed number per family	Phenotype for LBW	Controlled

^a^Sampling of larvae for phenotyping randomly over the whole population or a fixed number per family (see Fig. 2)

^b^Selection of adult flies random or based on phenotype for larval body weight (see Fig. 2)

^c^Mating in a group or controlled (see Fig. 2)

^d^LBW = larval body weight

### Breeding schemes

In all breeding schemes, a base population of 1500 unrelated males and 1500 unrelated females was simulated. All base animals were phenotyped, the 400 heaviest larvae were preselected, and 200 females and 100 males were selected for breeding according to each breeding scheme design (Fig. 2). In subsequent generations, the breeding scheme shown in Fig. 2 was followed. In selection step 1 (Fig. 2), larvae were sampled for phenotyping, either randomly across the population (Pop- scenarios) or a fixed number was phenotyped for LBW per full sib family (Fam- scenarios) and the 400 heaviest larvae were preselected (step 2). In the preselection step, the sex of an individual was not known. Because female BSF are on average heavier than males as larvae [7, 9], females were simulated to be heavier on average by an amount such that the preselection ratio of 3 males:7 females was achieved in the breeding schemes with 3000 phenotyped and 400 preselected larvae, based on experience in practice (Personal communication L.S. Hansen, Aarhus University). This preselection ratio was achieved by simulating the average female weight to be 0.9 genetic standard deviation units higher than the population mean, which implies that the preselected ratio differed between breeding schemes with different numbers of phenotyped and preselected larvae. Preselected larvae were either housed in a group or individually, until adult eclosure. At the adult stage, individuals were sexed. When housed in a group, it was not possible to identify which flies were the heaviest as larvae. Thus, the 200 females and 100 males necessary for breeding were randomly selected from the 400 previously preselected individuals (-Rand- scenarios). When housed individually, it was possible to select adult flies on their phenotype for LBW and they were also sexed at this point (-Phen- scenarios). As a result, the 200 females and 100 males necessary for breeding could be selected based on their phenotype for LBW (step 3). In step 4, mating occurred either in a group with all selected males and females together (-Group scenarios) or controlled, with two random females per male (-Cntrl scenarios). When mating occurred in a group, a randomly selected fraction of the males mated 3 times (33%), another fraction mated 2 times (34%), and the last mated 1 time (33%). Every generation, approximately 40,000 eggs were produced, based on 200 offspring per female (lower limit of the range reported by [1]).

### Phenotyped and preselected larvae

Several additional factors were varied for each of the 8 breeding schemes presented in Table 1:

- The number of individuals that were phenotyped for LBW was 400, 1000, or up to 10,000 in steps of 1000 (Fig. 2, step 1). The number of preselected individuals was 400 regardless of the number of phenotyped individuals.
- The number of individuals that were preselected based on LBW was 300, 350, 400, 500, or 600 (Fig. 2, step 2). This was the number of individuals that was kept alive before the selection of breeding animals, which was always 200 females and 100 males. The number of phenotyped individuals was 3000 regardless of the number of preselected individuals.

### Statistical analyses

Rates of genetic gain were calculated by linear regression of additive genetic levels on time. Rates of inbreeding were calculated by linear regression of log-transformed levels of inbreeding on time. Only time steps 5 to 10 were utilized for these analyses. To identify the optimal breeding strategy, the breeding programs were ranked, first based on genetic gain in LBW and second based on low rates of inbreeding per generation. Differences in rates of genetic gain and inbreeding between breeding programs were tested using one-way ANOVA, followed by a post-hoc test for pairwise comparisons. Breeding programs which did not significantly differ in genetic gain were grouped together. The breeding program with the lowest inbreeding within the group of breeding programs with the highest genetic gain was considered the best. All breeding schemes were compared the program with 3000 larvae phenotyped for LBW and 400 larvae preselected. In addition, within each breeding scheme, comparisons were made for different numbers of phenotyped and preselected larvae. All statistical analyses were performed using the R statistical software v.4.3.1 [18].

## Results

### Breeding schemes

Selecting adult flies based on their phenotype for LBW (-Phen- scenarios) significantly (p < 0.05) increased genetic gain compared to scenarios with random selection of adult flies (-Rand- scenarios; Table 2). For example, genetic gain in LBW for scenario Pop-Phen-Group was 7% higher compared to genetic gain for scenario Pop-Rand-Group. Mating all breeding animals in a group (-Group scenarios) realized a higher rate of inbreeding compared to controlled mating (-Cntrl scenarios), although this difference was not significant for the Pop- scenarios (random sampling of larvae for phenotyping across the population). Fixing the number of phenotyped larvae per family (Fam- scenarios) significantly increased rates of inbreeding compared to the Pop- scenarios.

**Table 2:** Effect of breeding scheme design^a^ on rates of inbreeding and genetic gain, in absolute values^b^ and relative to breeding scheme Pop-Rand-Group

	Genetic gain LBW^c^			Inbreeding		
Breeding scheme	Absolute	Relative (%)	Significance^d^	Absolute (%)	Relative (%)	Significance
Pop-Rand-Group	0.77	100	a	0.36	100	ab
Pop-Rand-Cntrl	0.77	100	a	0.34	95	a
Pop-Phen-Group	0.83	107	bc	0.39	110	c
Pop-Phen-Cntrl	0.83	108	c	0.37	104	bc
Fam-Rand-Group	0.77	100	a	0.51	143	e
Fam-Rand-Cntrl	0.78	100	a	0.49	136	d
Fam-Phen-Group	0.82	106	b	0.60	166	g
Fam-Phen-Cntrl	0.83	108	c	0.57	159	f

^a^For each breeding scheme, phenotyping was either random across the population (Pop-) or performed for a fixed number of individuals per family (Fam-), selection of adults was either random from preselected candidates (-Rand-) or based on phenotypic record for LBW (-Phen-) and mating of breeding animals either happened in a group (-Group) or at a 1:2 male:female ratio (-Cntrl). All breeding schemes included 3000 larvae phenotyped for LBW and 400 individuals preselected based on LBW

^b^Inbreeding is expressed as rate of inbreeding per generation in %, genetic gain is expressed in rate of change in genetic standard deviation units per generation. Rate of inbreeding and genetic gain were calculated using generations 5–10

^c^LBW = Larval body weight

^d^Different letters within a column indicate significant differences between breeding schemes

### Number of phenotyped larvae

**Fig. 3:**
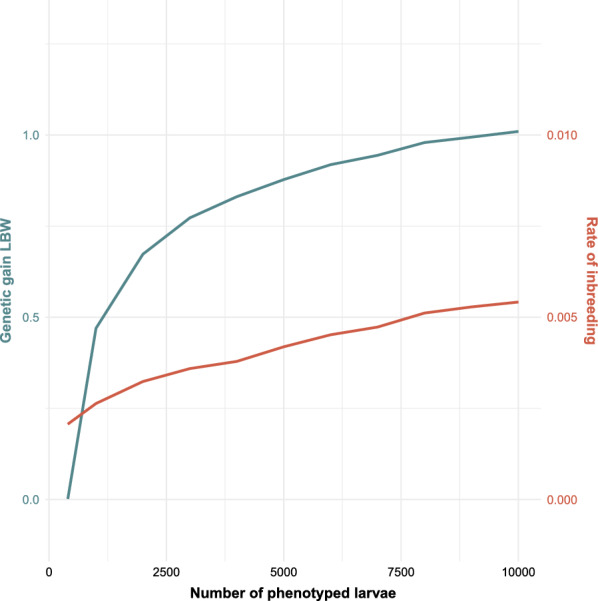
Effect of number of phenotyped larvae on the rate of genetic gain (in genetic standard deviation units per generation, in blue) and inbreeding per generation (in red), in breeding scheme Pop-Rand-Group. The number of preselected larvae was 400. *LBW* Larval body weight

Phenotyping more larvae when the number of preselected larvae was fixed at 400 increased rates of genetic gain and inbreeding, as can be seen for the breeding scheme Pop-Rand-Group (Fig. 3). When phenotyping only 400 larvae, genetic gain was very low for all breeding schemes and was 0 for the -Rand- breeding schemes [see Additional file 1 Table S1]. The effect of phenotyping more larvae on genetic gain was largest at low numbers of phenotyped larvae. For all breeding schemes, the highest genetic gain was achieved when phenotyping 10,000 larvae; 22 to 37% higher relative to phenotyping 3000 larvae. Genetic gain when phenotyping 10,000 larvae was not significantly different from genetic gain when phenotyping 9000 larvae, for all breeding schemes. When increasing the number of phenotyped larvae, the rate of inbreeding increased more in the Fam- scenarios than for the Pop- scenarios [see Additional file 2 Table S2].

The effect of increasing the number of phenotyped larvae in breeding schemes Pop-Rand-Group and Pop-Phen-Cntrl is shown in Table 3. For breeding scheme Pop-Rand-Group, phenotyping 1000 larvae more increased genetic gain to similar rates as for breeding scheme Pop-Phen-Cntrl with phenotyping 1000 larvae less. For example, phenotyping 4000 larvae for the Pop-Rand-Group scheme resulted in a genetic gain of 0.83 genetic standard deviation units, while phenotyping 3000 larvae for the Pop-Phen-Cntrl scheme resulted in the same genetic gain and a similar rate of inbreeding per generation.

**Table 3:** Effect of number of phenotyped^a^ larvae on rates of genetic gain and inbreeding for breeding schemes Pop-Rand-Group and Pop-Phen-Cntrl

No. phenotyped	Pop-Rand-Group				Pop-Phen-Cntrl			
	Genetic gain^b^		Inbreeding^c^		Genetic gain		Inbreeding	
	Absolute	Significance^d^	Absolute (%)	Significance	Absolute	Significance	Absolute (%)	Significance
400	0.00	a	0.21	a	0.21	a	0.21	a
1000	0.47	b	0.26	b	0.56	b	0.26	b
2000	0.67	c	0.32	c	0.73	c	0.34	c
3000	0.77	d	0.36	d	0.83	d	0.37	cd
4000	0.83	e	0.38	d	0.89	e	0.40	d
5000	0.88	f	0.42	e	0.93	f	0.43	e
6000	0.92	fg	0.45	f	0.97	fg	0.49	f
7000	0.94	gh	0.47	f	0.99	gh	0.50	fg
8000	0.98	hi	0.51	g	1.01	hi	0.50	fg
9000	0.99	i	0.53	gh	1.04	ij	0.53	g
10,000	1.01	i	0.54	h	1.06	j	0.58	h

^a^The number of preselected larvae was 400

^b^Genetic gain in larval body weight is expressed in rate of change in genetic standard deviation units per generation. The first four generations were omitted for the calculations of rate of inbreeding and genetic gain

^c^Inbreeding is expressed as rate of inbreeding per generation in %

^d^Different letters within a column indicate significant differences between no. phenotyped

### Number of preselected larvae

**Fig. 4:**
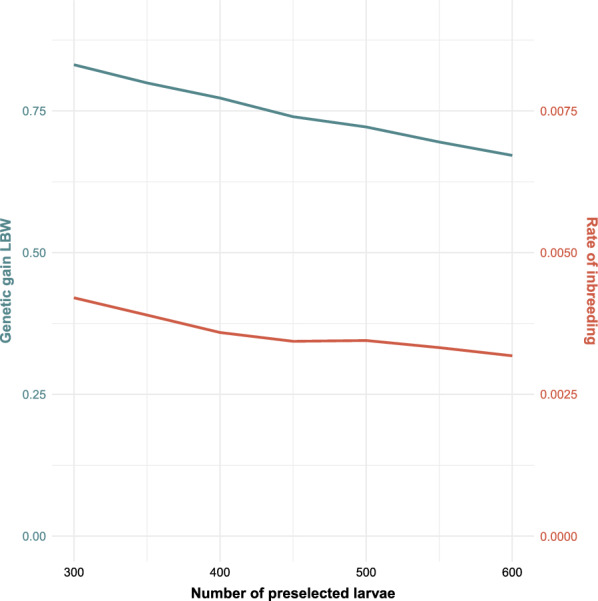
Effect of number of preselected larvae on the rate of genetic gain (in genetic standard deviation units per generation, in blue) and inbreeding per generation (in red), in breeding scheme Pop-Rand-Group. The number of phenotyped larvae was 3000. *LBW* Larval body weight

**Fig. 5:**
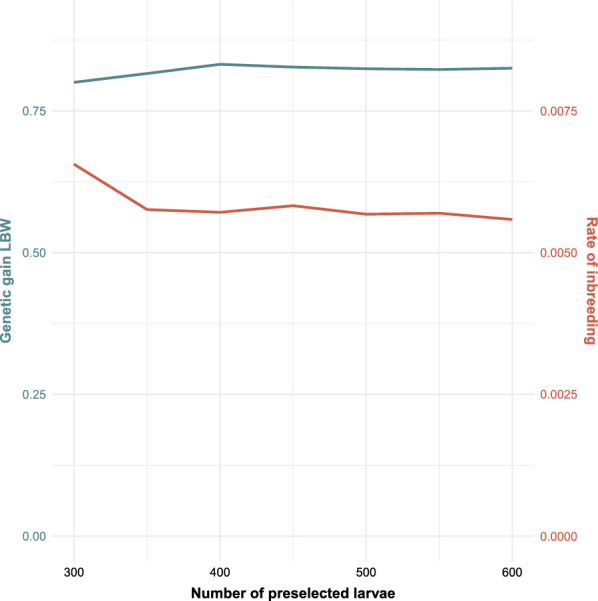
Effect of number of preselected larvae on the rate of genetic gain (in genetic standard deviation units per generation, in blue) and inbreeding per generation (in red), in breeding scheme Fam-Phen-Cntrl. The number of phenotyped larvae was 3000. *LBW* Larval body weight

The effect of the number of phenotyped larvae that were preselected and kept alive until adult eclosion differed between the -Rand- and -Phen- breeding schemes (Figs. 4, 5). In the -Rand- breeding schemes, increasing the number of preselected larvae decreased rates of genetic gain and inbreeding [see Additional file 3 Table S3 and Additional file 4 Table S4] but had only a slight effect on rates genetic gain and rate of inbreeding for the -Phen- breeding schemes. For the -Phen- breeding schemes, the rate of inbreeding increased slightly when less than 400 larvae were preselected.

### Sex ratio

The sex ratio of preselected males and females was set to 3:7 in the breeding schemes with 3000 phenotyped and 400 preselected larvae. Increasing the number of phenotyped larvae decreased the male:female sex ratio and increasing the number of preselected larvae slightly increased the male:female sex ratio of preselected larvae [see Additional file 5 Fig. S5 and Additional file 6 Fig. S6.].

## Discussion

Our results highlight that breeding programs in which the adult fly is selected based on their larval phenotype (-Phen- scenarios) and in which phenotyped larvae are sampled randomly from the population (Pop- scenarios) are most suited for BSF in terms of realizing most genetic gain with low rates of inbreeding. Controlled mating had only a minor effect on the rate of inbreeding but would significantly increase the workload related to establishing mating pairs. Thus, the recommended breeding program is Pop-Phen-Group. In addition, preselecting 400 larvae ensured enough male and female selection candidates for the scenarios evaluated in this study [see Additional file 6 Fig. S6]. Phenotyping more than 4000 larvae resulted in a proportion of males being preselected that was too low [see Additional file 5 Fig. S5] and is therefore discouraged. The reason for this is that female larvae are on average heavier than males and at this selection step, the sex of an individual is unknown. Phenotyping more larvae when preselection is based on LBW results in a more intense selection, thus resulting in less males being selected. Therefore, an additional recommendation is that the number of preselected animals needs to be calculated based on the sexual dimorphism of LBW to ensure enough male selection candidates.

### Selecting adults on larval phenotypes

All -Phen- breeding schemes resulted in significantly higher genetic gain compared to the -Rand- breeding schemes. Being able to select adult flies on LBW was the only element of the breeding schemes simulated in this study that significantly affected genetic gain when comparing breeding schemes with 3000 phenotyped and 400 preselected larvae. The ability to connect juvenile phenotypes to adult individuals allows for an extra selection step, instead of selecting randomly from among the preselected individuals, thereby increasing the genetic superiority of the breeding animals. However, the rate of inbreeding also increased when switching from a -Rand- to -Phen- breeding scheme, by 0.03 to 0.08%, because the probability of selecting siblings or half-siblings increased in the second selection step because relatives have similar phenotypes. The manual labor associated with selecting adults based on LBW is mainly related to need to house all preselected larvae in individual containers, i.e., they need to be prepared and labelled. In addition, individual housing may affect survival or other life history traits such as time until adult eclosion, since adult eclosion is influenced by environmental cues from other pupae. An advantage of housing larvae individually until adult eclosion is that they are easier to sex than flies that are housed in a group, which requires catching them. In addition, if variation in time until adult eclosion is large, housing larvae individually means preventing unwanted mating between early emerging flies. Thus, in a breeding program for BSF, being able to select breeding animals on their phenotype for LBW is very important for genetic gain, but may have some practical implications that are not considered in this study. In the future, computer vision and machine learning algorithms may enable accurate prediction of the sex of larvae, thereby omitting the necessity of the preselection step [19]. This would impact the optimal design of the breeding program.

### Sampling for phenotyping

Larvae were either randomly sampled from the entire population for phenotyping for LBW (Pop- scenarios), or a fixed number of larvae were sampled from each full sib family (Fam- scenarios). Fixing the number of phenotyped offspring per family requires larvae to be housed in family groups, which introduces a common environmental effect. Rate of inbreeding was 0.1 to 0.20% higher in the Fam- scenarios than in the Pop- scenarios. A high common environmental effect, as is often the case in insect production, increases the between family variance. Larvae in the same family share the same environment, which has a large effect on the phenotype of all members of the family when housed together. This increases the likelihood of related individuals to be selected, which increases the rate of inbreeding in a mass selection breeding program. In contrast, housing all larvae from one generation together results in common environmental effects to be the same for all and thus a lower chance of selecting related individuals.

### Mating

Mating all breeding animals semi-randomly in a group (-Group scenarios) compared to controlled mating with two females per male (-Cntrl scenarios) increased inbreeding because males could mate 1, 2, or 3 times in the former, causing unequal contributions to the next generation. Unequal contribution of sires highly impacts the rate of inbreeding and effective population size, see e.g. [20]. Unequal male contributions are to be expected when mating is in a group, but the exact reproductive capacity of BSF males and the distribution of how many males mate more often than others is unknown. The simulated design with 33% percent of the males mating once, 34% mating twice, and 33% percent mating three times is an assumption, created to simulate the effect of unequal male contributions. The results for the Pop- scenarios showed no significant differences in rates of inbreeding between the -Group and -Cntrl scenarios, implicating that the effect of these unequal male contributions on rates of inbreeding and genetic gain were limited. Controlled mating greatly increases manual labor since all adults need to be collected when eclosed, sexed, and then housed with one male and two females per container. In addition, it is not known whether controlled mating with only one male and two females is enough for reproductive success. For example, males demonstrate lekking behavior in wild populations [21] and restricting access to other breeding animals could decrease mating success to a point where obtaining the required number of families in the breeding scheme may fail. Therefore, controlling mating is not recommended in a BSF breeding program based on mass selection. However, the practical implications must be further studied to be able to quantify the full effect of this breeding program element.

### Number of phenotyped larvae

Not surprisingly, the number of phenotyped larvae had a large effect on genetic gain. Phenotyping more individuals increases selection intensity, resulting in the average weight of (pre)selected animals to be higher, leading to greater genetic gain. The effect of phenotyping more larvae on genetic gain was very high at smaller numbers phenotyped, but slowly decreased at higher numbers of phenotyped larvae, consistent with the effect of sample size on selection intensity. Phenotyping more than 9000 larvae did not lead to significantly higher genetic gain in any of the breeding schemes and, therefore, the extra workload related to this is not worthwhile.

The sex ratio of preselected males and females was set to 3:7 in the breeding schemes with 3000 phenotyped and 400 preselected larvae. Phenotyping more or fewer than 3000 larvae affected the proportion of selected males in the preselection step [see Additional file 5 Fig. S5]. When 400 larvae where phenotyped, all had to be preselected and thus the sex ratio was 1:1, equal to the sex ratio in the phenotyped group. This always ensured enough male selection candidates but, due to the stochastic nature of the sex ratio, fewer than 200 female selection candidates could be available. This meant that not all selected males could mate (given the assigned mating capacities in the simulation), resulting in fewer than 100 males and 200 females being used as breeding animals. In contrast, phenotyping more larvae resulted in a lower proportion of males being preselected based on weight, resulting in a lack of male candidates. For example, phenotyping 10,000 larvae resulted in only 24% males being preselected [see Additional file 5 Fig. S5], which caused an increase in the rate of inbreeding. Therefore, the number of phenotyped and preselected larvae needs to be carefully calculated based on the expected difference in average weight between the sexes to ensure enough male and female selection candidates are available.

### Number of preselected larvae

The number of preselected larvae had a very different effect on rates of genetic gain and inbreeding depending on whether adult flies were selected at random or based on their phenotype for LBW. When larvae were housed individually after the preselection step and adult flies were selected based on their phenotype for LBW, the function of the preselection step was merely to ensure that there were enough males and females for breeding, since individuals can only be sexed as adult flies and, therefore, the sex ratio was skewed when selecting larvae based on their phenotype for LBW. Enough males were expected to be selected when preselecting 400 larvae (based on 3000 phenotyped larvae), considering the skewed sex ratio that favored females and randomness caused by the stochastic nature of the simulation. This was also observed for breeding scheme Fam-Phen-Cntrl (Fig. 5), i.e. preselecting fewer than 400 larvae caused an increase in inbreeding. The reason for this was a lack of male selection candidates [see Additional file 6 Fig. S6]. Preselecting more than 400 larvae did not affect inbreeding nor genetic gain. In the breeding schemes in which adult flies could not be selected based on their phenotype for LBW, breeding flies were chosen at random from the group of preselected larvae. In this case, the larger the group of preselected larvae was, the lower was the average weight of these larvae and thus the lower was the average weight of the randomly chosen breeding flies. Therefore, in a practical breeding program it is highly recommended for the number of preselected larvae to be based on the number of required breeding flies, considering the skewed sex ratio when selecting on weight, and not to exceed this number.

### Practical feasibility

Implementing the suggested strategies from this study in a real-life breeding program increases the manual workload. Quantifying the manual labor induced by the different elements of the breeding schemes that were simulated was beyond the scope of this study, but it is a given that phenotyping is one of the major labor-intensive costs related to running an insect breeding program. Automated phenotyping techniques have the potential to substantially decrease manual labor requirements [15]. Running a more labor-intensive breeding program and decreasing phenotyping efforts will result in lower rates of inbreeding and comparable rates of genetic gain to running a less labor-intensive breeding program with increased phenotyping efforts (Table 3). Depending on labor requirements, an insect producer can decide which breeding program is better.

Simulation studies cannot include all practical aspects that are encountered when running a real-life breeding program. For example, this simulation assumed no mortality and completely successful matings, which influences the number of larvae available for phenotyping and the number of male and female breeding animals needed to successfully run the breeding program. Another aspect that was excluded from this study is synchronization of reproduction in order to minimize differences in age and ensure mating compatibility of selection candidates. In the simulation, all eggs hatched at the same time and thus all viable larvae were available for phenotyping at the same time. This may not be the case. However, eggs can be cold stored to synchronize hatching of eggs. When there is large variation in time from hatching to prepupae, the chance of selecting many larvae from one family and none from another will most likely be higher, especially since development time is moderately heritable [16]. This can increase relatedness in the pool of selection candidates. Adult flies will eclose over several days and adults that are too dissimilar in age will not be mating compatible. In practice this means that not all selected breeding animals will be able to mate with each other, which could decrease the selection intensity and consequently genetic gain. The number of preselected individuals must be adjusted accordingly to ensure enough breeding animals. Alternatively, cold storage of pupae can be applied to delay adult eclosion [22]. Finally, the genetic gain obtained in a breeding nucleus is only transferable to the production population if the environments are not drastically different. Running an advanced breeding program with family housing could introduce discrepancies between the breeding and production environments, resulting in undesirable genotype-by-environment interactions. It is crucial to reduce such differences to ensure that the genetic progress is replicated in the production population.

### (Un)favorable effects on other traits

The reason for only including LBW in the simulation was that we did not have access to genetic parameter estimates for other traits that might be of interest from a breeding perspective. Without sound estimates for genetic correlations and heritabilities, it is impossible to include other traits in the simulation. However, it is very likely that other economically important traits are correlated to the trait simulated to be under selection in this study. For example, [16] found unfavorable phenotypic correlations between LBW and development time in BSF. Mass selection complicates selection of breeding animals for multiple traits, especially when traits are measured in different life stages. However, it is very important to track other traits in a breeding program in order to study the effect of selection on genetic gain in other traits. Directional selection for LBW might cause unfavorable genetic changes in traits related to development or fertility. If this is the case, it is important to adjust the selection method to minimize the unfavorable effects. Implementing breeding value estimation with phenotypes for multiple traits and selecting on an index would enable breeders to genetically change multiple traits in the desired direction. The simulated breeding scheme Fam-Phen-Cntrl would enable the implementation of index selection with estimation of breeding values because it allows the pedigree of selection candidates to be tracked. In this case, housing family groups in at least two different environments is crucial in order to be able to separate the additive genetic effect from common environmental effects.

## Conclusions

A breeding program in which sampling for phenotyping is random across population, in which adult flies can be selected based on their larval phenotypes, and in which animals mate in a group is recommended for BSF, considering their positive effects on rates of genetic gain and inbreeding. Phenotyping a fixed number of offspring per family requires group housing, which introduces common environment effects, which greatly increase inbreeding. Housing offspring from all families together avoids the introduction of common environment effects at the family level. The option to select adult flies based on larval phenotypes allows for multistage selection, which greatly increases genetic gain. Compared to group matings, controlled matings did not have a profound effect on the rate of inbreeding. In practical production systems, however, the effect of group mating on inbreeding will depend on variation of the contributions of individual males to the next generation, which is not known at present. Finally, the number of phenotyped and preselected larvae should be calculated based on the expected sex difference in average LBW to ensure enough male selection candidates are preselected.

## Supplementary Information

Additional file 1: Title: Effect of number of phenotyped larvae on genetic gain per generation for all breeding schemes. The number of preselected larvae was 400.Description: Table showing genetic gain per generation for different number of phenotyped larvae per breeding scheme, for 400 preselected larvae.

Additional file 2: Title: Effect of number of phenotyped larvae on rate of inbreeding per generation for all breeding schemes. The number of preselected larvae was 400. Description: Table showing rate of inbreeding per generation for different number of phenotyped larvae per breeding scheme, for 400 preselected larvae.

Additional file 3: Title: Effect of number of preselected larvae on genetic gain per generation for all breeding schemes. The number of phenotyped larvae was 3000. Description: Table showing genetic gain per generation for different number of preselected larvae per breeding scheme, for 3000 phenotyped larvae.

Additional file 4: Title: Effect of number of preselected larvae on rate of inbreeding per generation for all breeding schemes. The number of phenotyped larvae was 3000. Description: Table showing rate of inbreeding per generation for different number of preselected larvae per breeding scheme, for 3000 phenotyped larvae.

Additional file 5: Title: Effect of number of phenotyped larvae on proportion of preselected males and number of selected males in breeding scheme Fam-Phen-Cntrl. The number of preselected larvae was 400. Description: Barplot showing the proportion of preselected males and number of selected males when increasing the number of phenotyped larvae.

Additional file 6: Title: Effect of number of preselected larvae on proportion of preselected males and number of selected males in breeding scheme Fam-Phen-Cntrl. The number of phenotyped larvae was 3000. Description: Barplot showing the proportion of preselected males and number of selected males when increasing the number of phenotyped larvae.

## Data Availability

Data will be available upon request.

## References

[1] van Huis A, Oonincx DGAB, Rojo S, Tomberlin JK. Insects as feed: house fly or black soldier fly? J Insects Food Feed. 2020;6:221–9.

[2] Sánchez-Muros MJ, Barroso FG, Manzano-Agugliaro F. Insect meal as renewable source of food for animal feeding: a review. J Clean Prod. 2014;65:16–27.

[3] van Huis A. Potential of insects as food and feed in assuring food security. Annu Rev Entomol. 2013;58:563–83. 23020616 10.1146/annurev-ento-120811-153704

[4] Veldkamp T, Van RK, Elissen H, Van WP, Van Der WR. Bioconversion of digestate, pig manure and vegetal residue-based waste operated by black soldier fly larvae, Hermetia illucens l. (Diptera: Stratiomyidae). Animals. 2021;11:3082. 34827814 10.3390/ani11113082 PMC8614437

[5] De Smet J, Wynants E, Cos P, Van Campenhout L. 2018. Microbial Community Dynamics during Rearing of Black Soldier Fly Larvae (Hermetia illucens) and Impact on Exploitation Potential. Appl Environ Microbiol 84:e02722-17. 10.1128/AEM.02722-17 10.1128/AEM.02722-17 PMC5930328 29475866

[6] Liu X, Chen X, Wang H, Yang Q, Ur Rehman K, Li W, et al. Dynamic changes of nutrient composition throughout the entire life cycle of black soldier fly. PLoS ONE. 2017;12:1–21. 10.1371/journal.pone.0182601 PMC5552164 28796830

[7] Tomberlin JK, Adler PH, Myers HM. Development of the black soldier fly (Diptera: Stratiomyidae) in relation to temperature. Environ Entomol. 2009;38:930–4. 19508804 10.1603/022.038.0347

[8] Tomberlin JK, Sheppard DC, Joyce JA. Selected life-history traits of black soldier flies (Diptera: Stratiomyidae) reared on three artificial diets. Ann Entomol Soc Am. 2002;95:379–86.

[9] Zhou F, Tomberlin JK, Zheng L, Yu Z, Zhang J. Developmental and waste reduction plasticity of three black soldier fly strains (Diptera: Stratiomyidae) raised on different livestock manures. J Med Entomol. 2013;50:1224–30. 24843926 10.1603/me13021

[10] Morales-Ramos JA, Kelstrup HC, Guadalupe Rojas M, Emery V. Body mass increase induced by eight years of artificial selection in the yellow mealworm (Coleoptera: Tenebrionidae) and life history trade-offs. J Insect Sci. 2019;19:4. 30822778 10.1093/jisesa/iey110 PMC6403474

[11] Bryant EH, Meffert LM. An analysis of selectional response in relation to a population bottleneck. Evolution. 1995;49:626–34. 28565137 10.1111/j.1558-5646.1995.tb02299.x

[12] Oldenbroek K, Waaij L van der. Chapter 8.1.1: Mass selection. In: Textbook animal breeding and genetics for BSc students. Centre for Genetic Resources. The Netherlands and Animal Breeding and Genomics Centre. 2015. https://wiki.groenkennisnet.nl/space/TAB/3738425/Chapter+8.1.1%3A+Mass+selection. Accessed 15 Nov 2023.

[13] Bentsen HB, Olesen I. Designing aquaculture mass selection programs to avoid high inbreeding rates. Aquaculture. 2002;204:349–59.

[14] Facchini E, Shrestha K, Van Den BE, Junes P, Sader G. Long-term artificial selection for increased larval body weight of Hermetia illucens in industrial settings. Front Genet. 2022;13:1–9. 10.3389/fgene.2022.865490 PMC9240604 35783268

[15] Laursen SF, Hansen LS, Bahrndorff S, Nielsen HM, Noer NK, Renault D, et al. Contrasting manual and automated assessment of thermal stress responses and larval body size in black soldier flies and houseflies. Insects. 2021;12:380. 33922364 10.3390/insects12050380 PMC8146041

[16] Bouwman AC, Nugroho JE, Wongso D, van Schelt J, Pannebakker BA, Zwaan BJ, et al. Genetic parameters of black soldier flies estimated in full sib design. In: Proceedings of 12th World Congress on Genetics Applied to Livestock Production: 3–8 July 2022; Rotterdam. 2022.

[17] Pedersen LD, Sørensen AC, Henryon M, Ansari-Mahyari S, Berg P. ADAM: A computer program to simulate selective breeding schemes for animals. Livest Sci. 2009;121:343–4.

[18] R: a language and environment for statistical computing. Vienna: R Foundation for Statistical Computing; 2021.

[19] Nawoya S, Ssemakula F, Akol R, Geissmann Q, Karstoft H, Bjerge K, Mwikirize C, Katumba A, Gebreyesus G. Computer vision and deep learning in insects for food and feed production: a review. Comput Electron Agric. 2024;216: 108503.

[20] Brown RC, Woolliams JA, McAndrew BJ. Factors influencing effective population size in commercial populations of gilthead seabream. Sparus aurata Aquaculture. 2005;247:219–25.

[21] Tomberlin JK, Sheppard DC. Lekking behavior of the black soldier fly (Diptera: Stratiomyidae). Fla Entomol. 2001;84:729–30.

[22] Deruytter D, Bellezza Oddon S, Gasco L, Coudron CL. Cold storage: a tool for delayed and stable black soldier fly (Hermetia illucens) pupae eclosion. In: Book of Abstracts of the 74th Annual Meeting of the European Federation of Animal Science; 2023.

