# Supplementary material for: The effect of phenotyping, adult selection, and mating strategies on genetic gain and rate of inbreeding in black soldier fly breeding programs
Source: Genet Sel Evol. 2024 Nov 4;56:71. doi: 10.1186/s12711-024-00938-y (PMC11533340; doi:10.1186/s12711-024-00938-y)
Supplement: Supplementary file 1 — Additional file 1: Title: Effect of number of phenotyped larvae on genetic gain per generation for all breeding schemes. The number of preselected larvae was 400.Description: Table showing genetic gain per generation for different number of phenotyped larvae per breeding scheme, for 400 preselected larvae. [file 12711_2024_938_MOESM1_ESM.docx]

| **No. phenotyped** | **Pop-Rand-Group** | **Pop-Rand-Cntrl** | **Pop-Phen-Group** | **Pop-Phen-Cntrl** | **Fam-Rand-Group** | **Fam-Rand-Cntrl** | **Fam-Phen-Group** | **Fam-Phen-Cntrl** |
| --- | --- | --- | --- | --- | --- | --- | --- | --- |
| 400 | 0.00 | 0.00 | 0.22 | 0.21 | 0.00 | 0.00 | 0.19 | 0.19 |
| 1000 | 0.47 | 0.47 | 0.56 | 0.56 | 0.47 | 0.48 | 0.56 | 0.56 |
| 2000 | 0.67 | 0.67 | 0.74 | 0.73 | 0.68 | 0.68 | 0.74 | 0.74 |
| 3000 | 0.77 | 0.77 | 0.83 | 0.83 | 0.77 | 0.78 | 0.82 | 0.83 |
| 4000 | 0.83 | 0.84 | 0.88 | 0.89 | 0.83 | 0.84 | 0.88 | 0.88 |
| 5000 | 0.88 | 0.88 | 0.92 | 0.93 | 0.87 | 0.87 | 0.91 | 0.92 |
| 6000 | 0.92 | 0.92 | 0.96 | 0.97 | 0.90 | 0.90 | 0.95 | 0.95 |
| 7000 | 0.94 | 0.95 | 0.99 | 0.99 | 0.93 | 0.94 | 0.97 | 0.97 |
| 8000 | 0.98 | 0.97 | 1.01 | 1.01 | 0.95 | 0.95 | 1.00 | 0.98 |
| 9000 | 0.99 | 1.00 | 1.04 | 1.04 | 0.97 | 0.96 | 1.01 | 1.01 |
| 10,000 | 1.01 | 1.02 | 1.05 | 1.06 | 0.99 | 0.99 | 1.02 | 1.02 |
