# Supplementary material for: The effect of phenotyping, adult selection, and mating strategies on genetic gain and rate of inbreeding in black soldier fly breeding programs
Source: Genet Sel Evol. 2024 Nov 4;56:71. doi: 10.1186/s12711-024-00938-y (PMC11533340; doi:10.1186/s12711-024-00938-y)
Supplement: Supplementary file 2 — Additional file 2: Title: Effect of number of phenotyped larvae on rate of inbreeding per generation for all breeding schemes. The number of preselected larvae was 400. Description: Table showing rate of inbreeding per generation for different number of phenotyped larvae per breeding scheme, for 400 preselected larvae. [file 12711_2024_938_MOESM2_ESM.docx]

| **No. phenotyped** | **Pop-Rand-Group** | **Pop-Rand-Cntrl** | **Pop-Phen-Group** | **Pop-Phen-Cntrl** | **Fam-Rand-Group** | **Fam-Rand-Cntrl** | **Fam-Phen-Group** | **Fam-Phen-Cntrl** |
| --- | --- | --- | --- | --- | --- | --- | --- | --- |
| 400 | 0.21% | 0.19% | 0.23% | 0.21% | 0.15% | 0.13% | 0.16% | 0.14% |
| 1000 | 0.26% | 0.24% | 0.27% | 0.26% | 0.26% | 0.24% | 0.30% | 0.29% |
| 2000 | 0.32% | 0.30% | 0.35% | 0.34% | 0.42% | 0.38% | 0.47% | 0.45% |
| 3000 | 0.36% | 0.34% | 0.39% | 0.37% | 0.51% | 0.49% | 0.60% | 0.57% |
| 4000 | 0.38% | 0.38% | 0.42% | 0.40% | 0.62% | 0.57% | 0.70% | 0.67% |
| 5000 | 0.42% | 0.40% | 0.47% | 0.43% | 0.70% | 0.69% | 0.78% | 0.77% |
| 6000 | 0.45% | 0.43% | 0.47% | 0.49% | 0.77% | 0.71% | 0.86% | 0.92% |
| 7000 | 0.47% | 0.44% | 0.51% | 0.50% | 0.85% | 0.86% | 1.02% | 0.93% |
| 8000 | 0.51% | 0.47% | 0.53% | 0.50% | 0.94% | 0.86% | 1.09% | 1.02% |
| 9000 | 0.53% | 0.48% | 0.56% | 0.53% | 0.96% | 0.95% | 1.13% | 1.17% |
| 10,000 | 0.54% | 0.55% | 0.57% | 0.58% | 1.06% | 1.01% | 1.16% | 1.17% |
