# Supplementary material for: The effect of phenotyping, adult selection, and mating strategies on genetic gain and rate of inbreeding in black soldier fly breeding programs
Source: Genet Sel Evol. 2024 Nov 4;56:71. doi: 10.1186/s12711-024-00938-y (PMC11533340; doi:10.1186/s12711-024-00938-y)
Supplement: Supplementary file 3 — Additional file 3: Title: Effect of number of preselected larvae on genetic gain per generation for all breeding schemes. The number of phenotyped larvae was 3000. Description: Table showing genetic gain per generation for different number of preselected larvae per breeding scheme, for 3000 phenotyped larvae. [file 12711_2024_938_MOESM3_ESM.docx]

| **No. preselected** | **Pop-Rand-Group** | **Pop-Rand-Cntrl** | **Pop-Phen-Group** | **Pop-Phen-Cntrl** | **Fam-Rand-Group** | **Fam-Rand-Cntrl** | **Fam-Phen-Group** | **Fam-Phen-Cntrl** |
| --- | --- | --- | --- | --- | --- | --- | --- | --- |
| 300 | 0.83 | 0.83 | 0.84 | 0.84 | 0.81 | 0.80 | 0.82 | 0.80 |
| 350 | 0.80 | 0.81 | 0.83 | 0.83 | 0.79 | 0.79 | 0.82 | 0.82 |
| 400 | 0.77 | 0.77 | 0.83 | 0.83 | 0.77 | 0.78 | 0.82 | 0.83 |
| 450 | 0.74 | 0.74 | 0.83 | 0.83 | 0.74 | 0.74 | 0.83 | 0.83 |
| 500 | 0.72 | 0.72 | 0.83 | 0.83 | 0.72 | 0.71 | 0.82 | 0.82 |
| 550 | 0.70 | 0.69 | 0.83 | 0.83 | 0.69 | 0.69 | 0.81 | 0.82 |
| 600 | 0.67 | 0.67 | 0.83 | 0.83 | 0.67 | 0.68 | 0.82 | 0.83 |
