# Supplementary material for: The effect of phenotyping, adult selection, and mating strategies on genetic gain and rate of inbreeding in black soldier fly breeding programs
Source: Genet Sel Evol. 2024 Nov 4;56:71. doi: 10.1186/s12711-024-00938-y (PMC11533340; doi:10.1186/s12711-024-00938-y)
Supplement: Supplementary file 4 — Additional file 4: Title: Effect of number of preselected larvae on rate of inbreeding per generation for all breeding schemes. The number of phenotyped larvae was 3000. Description: Table showing rate of inbreeding per generation for different number of preselected larvae per breeding scheme, for 3000 phenotyped larvae. [file 12711_2024_938_MOESM4_ESM.docx]

| **No. preselected** | **Pop-Rand-Group** | **Pop-Rand-Cntrl** | **Pop-Phen-Group** | **Pop-Phen-Cntrl** | **Fam-Rand-Group** | **Fam-Rand-Cntrl** | **Fam-Phen-Group** | **Fam-Phen-Cntrl** |
| --- | --- | --- | --- | --- | --- | --- | --- | --- |
| 300 | 0.42% | 0.43% | 0.44% | 0.43% | 0.62% | 0.64% | 0.65% | 0.66% |
| 350 | 0.39% | 0.37% | 0.41% | 0.36% | 0.56% | 0.54% | 0.61% | 0.58% |
| 400 | 0.36% | 0.34% | 0.39% | 0.37% | 0.51% | 0.49% | 0.60% | 0.57% |
| 450 | 0.34% | 0.33% | 0.40% | 0.35% | 0.49% | 0.47% | 0.61% | 0.58% |
| 500 | 0.34% | 0.32% | 0.39% | 0.36% | 0.47% | 0.45% | 0.59% | 0.57% |
| 550 | 0.33% | 0.30% | 0.38% | 0.36% | 0.43% | 0.40% | 0.58% | 0.57% |
| 600 | 0.32% | 0.29% | 0.40% | 0.35% | 0.41% | 0.40% | 0.61% | 0.56% |
