# Supplementary figures and images for: The effect of phenotyping, adult selection, and mating strategies on genetic gain and rate of inbreeding in black soldier fly breeding programs
Source: Genet Sel Evol. 2024 Nov 4;56:71. doi: 10.1186/s12711-024-00938-y (PMC11533340; doi:10.1186/s12711-024-00938-y)

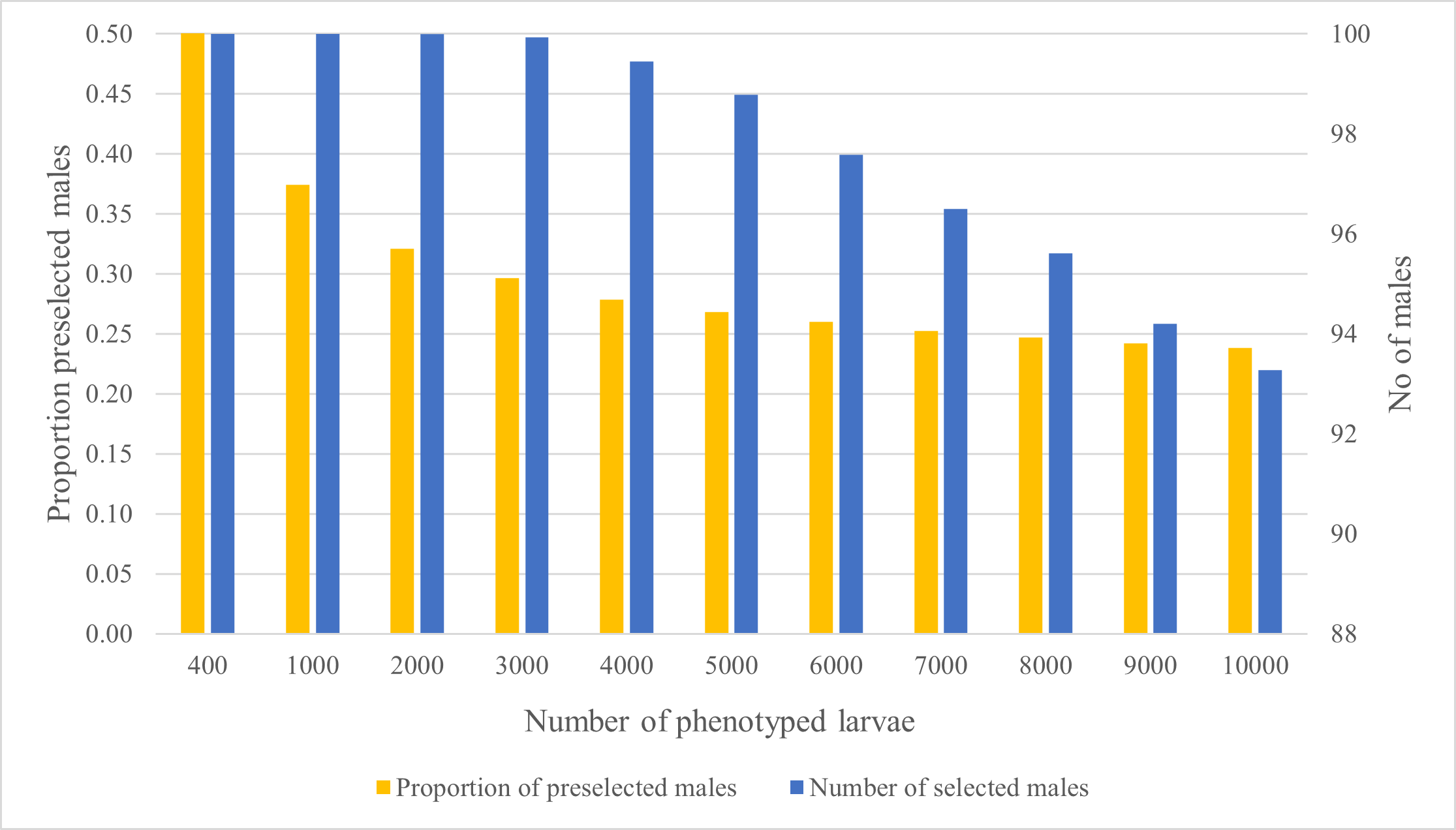

Supplement: Supplementary file 5 — Additional file 5: Title: Effect of number of phenotyped larvae on proportion of preselected males and number of selected males in breeding scheme Fam-Phen-Cntrl. The number of preselected larvae was 400. Description: Barplot showing the proportion of preselected males and number of selected males when increasing the number of phenotyped larvae. [file 12711_2024_938_MOESM5_ESM.png]

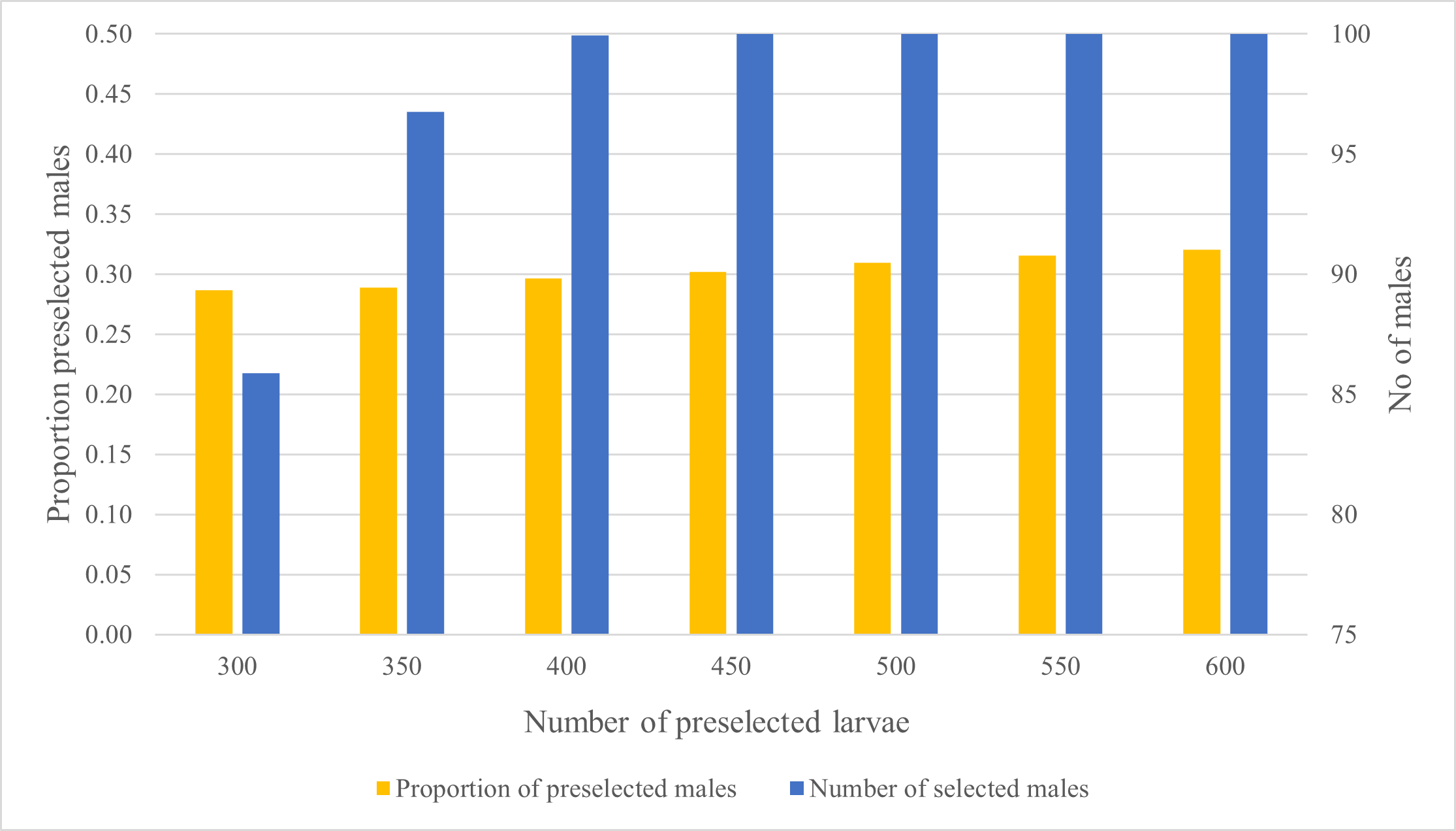

Supplement: Supplementary file 6 — Additional file 6: Title: Effect of number of preselected larvae on proportion of preselected males and number of selected males in breeding scheme Fam-Phen-Cntrl. The number of phenotyped larvae was 3000. Description: Barplot showing the proportion of preselected males and number of selected males when increasing the number of phenotyped larvae. [file 12711_2024_938_MOESM6_ESM.png]
